# The synergistic role of virtual coaching with simulation‐based mastery learning for upper endoscopy

**DOI:** 10.1002/deo2.317

**Published:** 2024-01-14

**Authors:** Tiffany Nguyen‐Vu, YungKa Chin, Carmel Malvar, Patricia Anne Cabral‐Prodigalidad, Mark De Lusong, Hasan Maulahela, Parit Mekaroonkamol, Andrew Ong, Angela Djajakusuma, Thomas Myint, Hilda Nurmalihah, Ravishankar Asokkumar, Carlos Francisco, Jesse Liu, Rungsun Rerknimitr, Amandeep Shergill, Silvia Sanduleanu, Tonya Kaltenbach, Roy Soetikno

**Affiliations:** ^1^ Department of Medicine University of California San Francisco USA; ^2^ Division of Gastroenterology and Hepatology San Francisco VA Medical Center San Francisco USA; ^3^ Department of Gastroenterology and Hepatology Singapore General Hospital Singapore Singapore; ^4^ Institute of Digestive and Liver Diseases St. Luke's Medical Center Taguig City Philippines; ^5^ Department of Gastroenterology University of the Philippines Philippine General Hospital Manila Philippines; ^6^ Division of Gastroenterology Department of Internal Medicine Faculty of Medicine University of Indonesia‐Cipto Mangunkusumo National General Hospital Jakarta Indonesia; ^7^ Department of Gastroenterology, Center of Excellence for Innovation and Endoscopy in Gastrointestinal Oncology Chulalongkorn University Bangkok Thailand; ^8^ Department of Gastroenterology and Hepatology California Pacific Medical Center San Francisco USA; ^9^ Division of Gastroenterology and Hepatology Maastricht University Medical Center Maastricht the Netherlands

**Keywords:** EGD, gastroenterology fellowship, endoscopy training, simulation‐based mastery learning, virtual coaching

## Abstract

**Introduction:**

Our simulation‐based mastery learning (SBML) curriculum, delivered in person, has been shown to successfully train novices in structured esophagogastroduodenoscopy (EGD). SBML with virtual coaching (VC) has the potential to improve the effectiveness and efficiency of endoscopy training and expand access to trainees from around the world. We share our observations conducting an EGD training course using SBML with VC.

**Methods:**

We conducted a 1‐week virtual SBML course for novice trainees across seven academic centers in the USA and Asia. The cognitive component was delivered using an online learning platform. For technical skills, a virtual coach supervised hands‐on training and local coaches provided assistance when needed. At the end of training, an independent rater assessed simulation‐based performance using a validated assessment tool. We assessed the clinical performance of 30 EGDs using the ASGE Assessment of Competency in Endoscopy tool. We compared the trainees’ scores to our cohort trained using in‐person SBML training using non‐inferiority t‐tests.

**Results:**

We enrolled 21 novice trainees (mean age: 30.8 ± 3.6 years; female: 52%). For tip deflection, the trainees reached the minimum passing standard after 31 ± 29 runs and mastery after 52 ± 37 runs. For structured EGD, the average score for the overall exam was 4.6 ± 0.6, similar to the in‐person cohort (4.7 ± 0.5, *p* = 0.49). The knowledge‐based assessment was also comparable (virtual coaching: 81.9 ± 0.1; direct coaching: 78.3 ± 0.1; *p* = 0.385). Over time, our novice trainees reached clinical competence at a similar rate to our historical in‐person control.

**Conclusions:**

VC appears feasible and effective for training novice gastroenterology trainees. VC allowed us to scale our SBML course, expand access to experts, and administer SBML simultaneously across different sites at the highest standards.

## INTRODUCTION

In endoscopy training, trainees typically learn through didactic lectures and apprenticeships. However, this method of procedural training is often unstandardized; it results in variable technical skills depending on the expertise of the supervising physician.^1^ Opportunities for training are also highly dependent on the caseload of individual institutions and the availability of training physicians.

Simulation‐based learning provides trainees with the opportunity to train procedural skills safely and effectively even before they begin performing endoscopy in patient settings. The few existing simulation‐based training programs usually focus exclusively on technical skills and rarely incorporate supporting educational curricula that emphasize competency and improvement in clinical practice.^2^, ^3^ Simulation‐based training coupled with mastery learning, a form of competency‐based education in which learners are required to meet or exceed a predetermined level of skill before completion of training,^4^ has been shown to improve clinical skills and reduce the risk of procedure‐associated injury for a variety of procedural skills. Although simulation‐based mastery learning (SBML) has been used in various surgical specialties, it is rarely used in endoscopy.^5^, ^6^, ^7^ Our research group has pioneered the use of SBML to facilitate the safe and efficient acquisition of basic and advanced procedural skills among endoscopy practitioners and trainees.^8^, ^9^, ^10^ Our experience with a structured esophagogastroduodenoscopy (EGD) SBML course for novice gastrointestinal (GI) fellows demonstrates the efficacy of an SBML course in helping trainees rapidly acquire upper endoscopy skills compared to traditional apprentice‐based training.^11^

During the coronavirus disease 2019 (COVID‐19) pandemic, many institutions temporarily halted routine endoscopic procedures to ensure patient and provider safety and to redeploy practitioners to other areas of immediate clinical need. These disruptions reduced caseload volume in endoscopy and inadvertently created a vacuum in educational and training activities for the trainees. Marasco et al reported that the lack of appropriate training for young trainees during the pandemic will not only widen the gap of deprived countries, but will also have a negative impact on them psychologically, resulting in burnout.^12^, ^13^ To adapt to the restrictions of the COVID‐19 pandemic, we delivered our training program using virtual training mechanisms. Virtual training can be used beyond the pandemic to supplement face‐to‐face training when travel opportunities and resources are not available.

There are no published studies showing the outcomes of virtual training for simulation‐based mastery learning in endoscopy. Herein, we share our observations from conducting an SBML EGD training course for novice fellows that was delivered using virtual coaching.

## METHODS

### Participants

**FIGURE 1:**
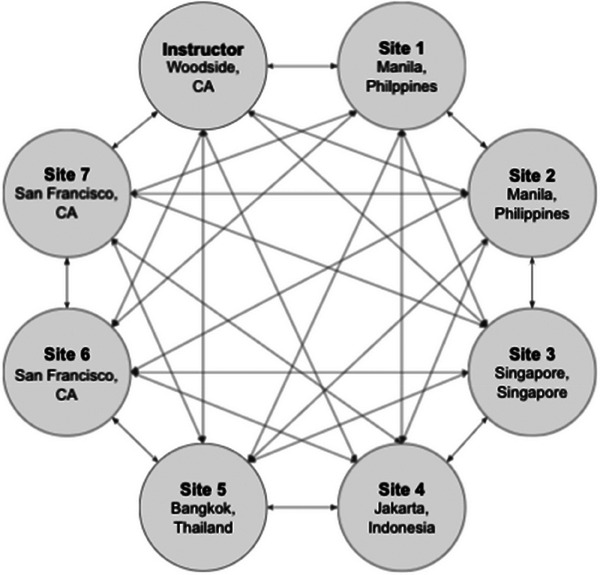
International esophagogastroduodenoscopy (EGD) course conducted mainly through virtual coaching.

We conducted an upper endoscopy training program for novice trainees (1st year GI fellows). The trainees were recruited from seven academic medical centers in San Francisco, US; Singapore, Singapore; Manila, Philippines; Jakarta, Indonesia; and Bangkok, Thailand (Figure 1). The course was conducted over a 1 week period (July 2020). The trainees were relieved of their clinical duties for the duration of the training program. We collected demographic information including age, gender, training track, dominant hand, and previous EGD and colonoscopy experience.

### Historical control

We compared our data to data obtained from our previous experience with a historical cohort (*n* = 6) that underwent the SBML curriculum through in‐person training at two sites in the United States and Singapore. The entire curriculum was delivered by in‐person coaches and with no virtual interventions. The core curriculum, which focused on standard EGD, was administered over the course of 1 week, similar to the course time frame for the virtual cohort (July 2019). The in‐person historical cohort was given the opportunity for an additional week of training that covered more advanced endoscopic techniques. Herein, we compare the results for only the EGD curriculum.

### Course curriculum

**FIGURE 2:**
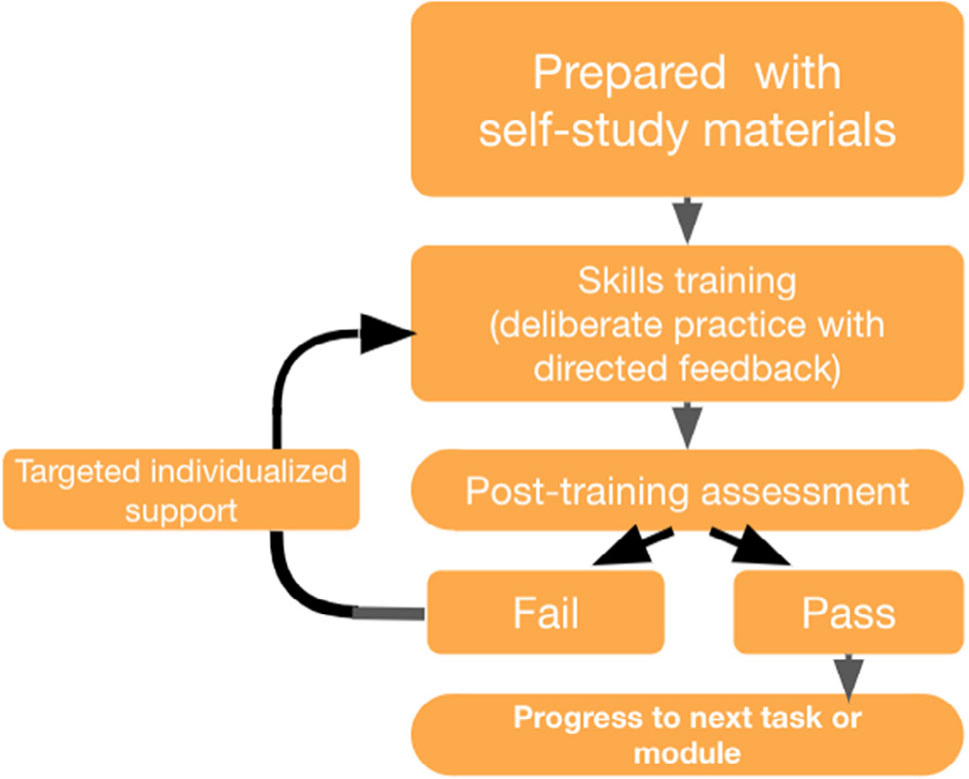
Structure of a standard simulation‐based mastery learning training course.

**FIGURE 3:**
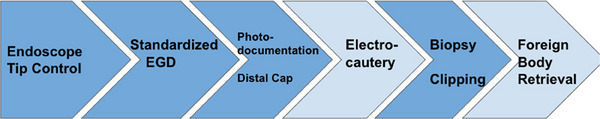
Course curriculum for the esophagogastroduodenoscopy (EGD) simulation‐based mastery learning training program delivered through virtual coaching.

We used the mastery learning framework to design our curriculum (Figure 2) which was described extensively in Nguyen‐Vu et al.^9^ Two expert‐level endoscopists determined the necessary elements to perform high‐quality upper endoscopy: appropriate diagnosis of common cancers and diseases, adequate endoscope tip control (fine motor movements), thorough mucosal examination of the upper GI tract, high‐quality photodocumentation, biopsy and clipping (Figure 3).

All students were given access to an online learning management system (Canvas, Salt Lake City, UT). Trainees completed the online modules concurrently with their simulation‐based training. For the written self‐assessments, they were required to achieve a score above 80% before attending the technical session.

To train endoscope tip control, we utilized a previously validated simulator that was designed to facilitate rapid acquisition of fine endoscopic motor movements.^14^ The trainee was required to target the stickers “A‐Z” using proper endoscope handling technique with only one hand controlling the endoscope. We collected the amount of time taken for each attempt to complete the simulator activity (completion time) and the number of attempts to reach competency (120 s) and mastery (100 s).

We aimed to teach the trainees how to perform a standardized EGD (a systematic process of performing a high‐quality mucosal examination of the upper GI tract). We emphasized that EGD should be performed deliberately and that every section of the upper GI tract must be examined completely. We used an upper GI simulator model and evaluated their skills using a previously validated assessment tool.^15^

Photodocumentation refers to the photographic screenshots taken from the endoscopy processor. We believe that high‐quality photodocumentation includes photos taken from distances close up, medium, and long‐view to achieve a robust understanding of the lesion (morphology, surface pattern, histology, etc.) and its location.

The trainees were introduced to the techniques of biopsy and clipping using the upper endoscopy simulator model. They were taught how to utilize one hand to control the endoscope and the other hand to control the tool. The trainees were instructed to biopsy or clip various regions of the stomach (i.e. antrum and fundus).

### Virtual coaching mechanism

**FIGURE 4:**
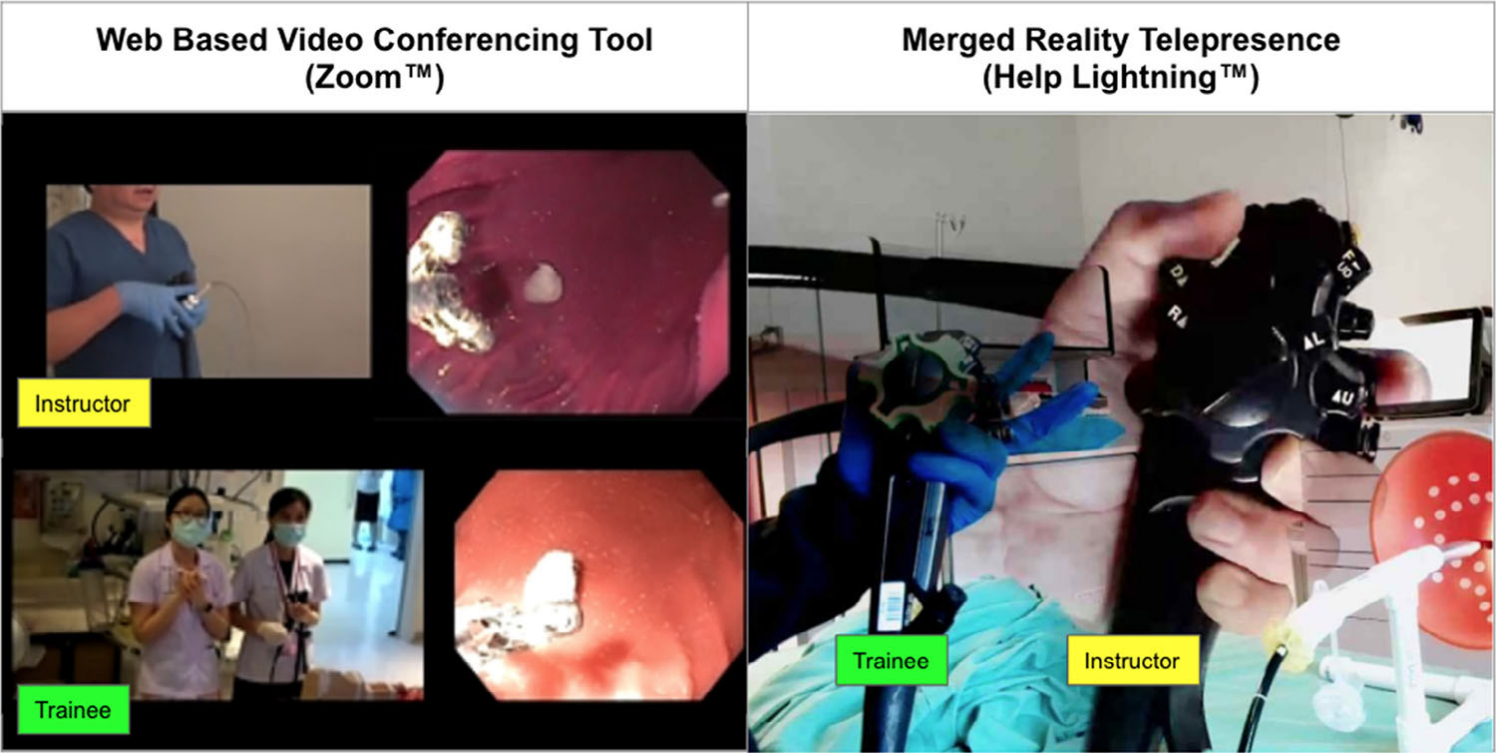
Virtual Coaching using Zoom (left) and Help Lightning (right).

We conducted daily virtual group lectures (Zoom, San Jose, CA, USA) to introduce the technique (1‐2 h). Then, we conducted small group sessions with approximately two trainees per group to provide more individual guidance and feedback (1–3 h). Both the trainees and instructors were able to view each other's endoscopy monitor as well as monitor their hands on the endoscope (Figure 4). We also used a merged reality software (Help Lightning, Birmingham, AL, USA) to provide real‐time synchronous feedback. This allowed for a more interactive experience, as the trainer was able to transpose an image onto the trainee's visual field, simulating a hands‐on training session. The small group sessions were moderated by virtual coaches (n = 3) who were all experienced practicing endoscopists (Roy Soetikno, YungKa Chin, and Mark De Lusong). At least one virtual coach was available on standby for any questions and additional guidance requested by the trainees.

### Minimum passing standards

The fellows were taught the techniques in order of increasing difficulty (Figure 2). The trainees were provided with iterative assessment and feedback throughout the training period. They were required to meet the set minimum passing standard (MPS) for each technique prior to moving on to the next topic.

The MPS to meet competency for endoscope handling, which was based on expert endoscopists’ performance, was to complete one full trial run on the simulator in under 120 s.^14^ After reaching competency, they continued to practice endoscope handling as a warm‐up for the following modules and aimed to reach mastery level (under 100 s). To meet competency for the EGD examination, trainees were required to score at least a “4” (Scale: 1‐Very Poor to 6‐Excellent) using a previously validated assessment tool by Neumann et al.^15^ We chose to use this tool as it was previously validated to assess simulation‐based EGD skills as opposed to clinical EGD skills.

On the final day of the program, the trainees then completed a final written assessment on Canvas. Four endoscopy educators collaborated to create the assessment which consisted of 20 multiple‐choice questions derived from their assigned articles and lectures. The questions assessed indication,^3^ management,^5^ anatomy,^5^ and diagnosis.^7^ The assessment has been previously evaluated to determine a significant difference between first‐year trainees and second‐ and third‐year trainees.^11^

Then, the trainees were assessed on their technical EGD skills on Zoom.^15^ The graders were experienced endoscopists who were not involved in their training. We assessed interrater reliability by having them rate 10 random EGD videos. We considered a kappa score >0.60 to be adequate.

### Feedback survey

After the trainees completed the final written and technical skills assessment, we delivered a feedback survey to the trainees and faculty regarding the strengths and weaknesses of the virtual coaching program. The survey consisted of 24 items that were open‐ended or rated on a 10‐point Likert scale.

### Clinical performance evaluation

One month after the course, we began evaluating the trainees’ performance in clinical EGD using the ASGE's Assessment of Competency in Endoscopy (ACE) Tool.^16^ The trainees were asked to collect the ACE tool for every EGD they performed or assisted in. The supervising endoscopist completed the ACE tool immediately after the trainee's procedure. We compared the scores to those from the historical cohort.

## RESULTS

We enrolled 21 novice trainees in our training program (Table 1). Most trainees had no prior experience performing EGD or colonoscopy in patients. All trainees (*n* = 21, 100%) completed the training program and met the MPS for endoscope handling, standard EGD examination, photo‐documentation, and biopsy. For endoscope handling analysis, we excluded four trainees in the virtual coaching cohort who had experience with the tip control simulator prior to the training program. The trainees reached the MPS for competency after 31.4 ± 29.1 attempts and mastery after 51.9 ± 36.7 attempts, similar to the historical cohort that had undergone the training with direct coaching (Table 2).

**TABLE 1:** Participant demographics.

	Virtual coaching (*n* = 21)	Direct coaching (*n* = 6)
**Gender**		
Male	10 (47.6)	6 (100)
Female	11 (52.4)	0 (0)
**Age (mean ± SD)**	30.8 ± 3.6 years	31.0 ± 2.7 years
**Number of prior EGDs**		
0	19 (90.4)	6 (100)
<5	1 (4.8)	0 (0)
5–10	1 (4.8)	0 (0)
>10	0 (0)	0 (0)
**Dominant hand**		
Right	17 (80.9)	5 (83.3)
Left	4 (19.1)	1 (16.7)
**Training track**		
Clinical	18 (85.7)	5 (83.3)
Research	0 (0)	1 (16.7)
Undifferentiated	3 (14.3)	0 (0)

Abbreviation: EGD, esophagogastroduodenoscopy.

**TABLE 2:** Comparison of trainee performance when taught using virtual coaching versus direct in‐person coaching.

	Virtual coaching cohort (*n* = 21)	Direct coaching cohort (*n* = 6)	*p*‐Value
**Knowledge‐based assessment**			
Mean % correct	81.9% + 8.9%	78.3% + 8.2%	*p* = 0.385
**Standard EGD examination**			
Overall mark for esophagus	5.1 ± 0.7	4.8 ± 0.8	*p* = 1.00
Overall mark for stomach	4.6 ± 0.5	5.2 ± 0.8	*p* = 0.06
Overall mark for duodenum	4.5 ± 0.8	5.0 ± 0.9	*p* = 0.52
General assessment of UGI tract	4.6 ± 0.6	4.7 ± 0.5	*p* = 1.00
	**Virtual coaching cohort (*n* = 17)**	**Direct coaching cohort (*n* = 6)**	***p*‐Value**
**Endoscope handling simulator**			
Attempts needed to reach competency	31.4 ± 29.1	32.5 ± 22.8	*p* = 0.93
Attempts needed to reach mastery	51.9 ± 36.7	38.2 ± 31.1	*p* = 0.42
	**Virtual coaching cohort (*n* = 6)**	**Direct coaching cohort (*n* = 6)**	***p*‐Value**
**Clinical EGD assessment**			
Mean ACE score for first 30 EGDs (overall hands‐on technique)	2.3 ± 0.8	2.2 ± 0.7	*p* = 0.25

Abbreviations: ACE, Assessment of Competency in Endoscopy; EGD, esophagogastroduodenoscopy; UGI, upper gastrointestinal.

For the overall knowledge‐based assessment, the mean score for the virtual coaching group was 81.9%±8.9%, similar to those trained through direct coaching only (78.3 ± 8.2%, *p* = 0.385).

For standard EGD, the mean score for the general assessment of the UGI tract was 4.6 + 0.6, which was similar to the scores of the historical cohort (4.7 + 0.5, *p* = 0.55; Table 2). The interrater reliability was adequate with a kappa score of 0.82. The average score (out of 6.0) for the overall mark was highest for the esophagus (5.1 ± 0.7), and lowest for the duodenum (4.5 ± 0.8).

### Feedback (trainees)

The average overall satisfaction rating for the course, including the online learning management system, virtual coaches, and simulation‐based practice sessions, was 9.3 ± 1.2 (out of 10) with 90% of the trainees indicating interest in attending similarly structured courses for other endoscopic techniques. The trainees reported high satisfaction with the realism of the virtual coaching set‐up (9.2 ± 0.95 out of 10), the helpfulness of their virtual coaches (9.49 ± 0.79), and the scheduling availability of their virtual coaches (9.22 ± 0.92). Twelve (57%) of the trainees indicated that the length of the course was appropriate, while 29% (*n* = 6) felt that it was too short and 14% felt it was too long.

### Feedback (faculty)

The local trainers and program directors rated the program highly. They were satisfied with the effectiveness of the overall training program (9.2 ± 0.4 out of 10.0) and found that the program was feasible for their site (9.0 ± 0.9). They felt that the bowl simulator was helpful in training endoscope handling (9.4 ± 0.5) and preparing novice fellows for EGD (9.1 ± 1.0). 89% of the respondents (*n* = 8) strongly agreed that they would like their site to participate in this program in the future.

They rated the realism of virtual coaching in simulating direct coaching as 8.8±0.8 and their satisfaction with the effectiveness of the virtual instructor as 9.1 ± 0.8. 22% of faculty (*n* = 2) strongly agreed that the virtual coaching mechanism was easy to set up while 67% (*n* = 6) agreed and 11% (*n* = 1) were neutral. After setting up, all trainers at least agreed that the mechanism was easy to use. A majority (89%, *n* = 8) at least agreed that virtual coaching can be used for training practicing endoscopists and would participate in a training program with virtual coaching to learn techniques like endoscopic mucosal resection or endoscopic suturing.

### Clinical performance evaluation

We collected 33 clinical EGD evaluations from six fellows (29%) in the virtual coaching group and 94 evaluations from six fellows (100%) from the direct coaching group. Collecting clinical evaluations from the supervising physicians was not feasible for most of our fellows due to the limited resources and procedures during the COVID‐19 pandemic.

For the first 30 clinical EGDs, the trainees in the virtual coaching group received similar scores as the direct coaching group (2.3 ± 0.8 vs. 2.2 ± 0.7; *p* = 0.25; Table 2).

## DISCUSSION

In the 1980s, a renowned educational psychologist, Benjamin Bloom, described a breakthrough learning method that combined mastery learning and intensive one‐on‐one mentoring.^17^ The students performed 98% better (on average) than those who learned using the traditional method. This increase in learning is massive; no other learning method could produce as much. The application of Bloom's learning method could have a significant impact on endoscopy. If it can be scaled in endoscopy, it can facilitate the dissemination of endoscopy knowledge and skills more effectively and efficiently, and could potentially close the disparity in the availability and quality of endoscopy. While mastery learning with one‐on‐one mentoring produces the most significant results, Bloom is concerned with the feasibility of such a system as one‐one‐one mentoring is logistically difficult for the trainer with additional responsibilities. Herein, we describe a proof of concept that we can use evidence‐based education principles with modern communication software to train upper endoscopy more efficiently and effectively.

In a prior study, we have shown that the direct coaching SBML program has resulted in significant improvement in trainee EGD skills compared to trainees who were taught with the traditional apprentice‐based training system.^11^ In this current study, virtual coaching appears to be as feasible and effective as direct coaching for the SBML program for novice gastroenterology trainees. The method of delivery for the training program (virtual coaching vs direct coaching) resulted in no significant difference in their cognitive assessments, simulation‐based EGD assessments, or the first 30 EGDs performed in clinical practice compared to trainees who underwent the program in person. To better illustrate the value of the virtual coaching training program, future studies will assess the difference in outcomes between trainees in the program compared to trainees who undergo traditional apprentice‐based training at the patient bedside.

A particular strength of the multicenter study, wherein the training programs were delivered across different centers in North America and Southeast Asia, is that we were able to demonstrate the feasibility and effectiveness of SBML with virtual coaching in endoscopy training despite differences in clinical and social settings. Our program allowed us to continue intensive endoscopy training during the COVID‐19 pandemic when routine procedures that trainees typically learn from were vastly canceled. Beyond the COVID‐19 pandemic, virtual coaching has the potential to bridge the training and quality gap worldwide, especially for those in rural and underserved areas. It may be worthwhile for certain trainees and trainers to consider this type of training when the opportunity to meet in person (due to financial, travel, or time restraints) is limited.

A limitation of our study is that we received limited clinical EGD evaluations from the virtual coaching group. The COVID‐19 pandemic heavily impacted and reduced clinical volume, thus limiting the trainees’ opportunities to perform EGDs in the clinic. Consequently, clinical follow‐up through ACE forms represented only a fifth of our virtual coaching cohort. In addition, our study included a small sample size for the control cohort. Due to the COVID‐19 pandemic restricting unnecessary gatherings, we could not continue recruitment for in‐person training. As such, we acknowledge the limited sample size when interpreting the assessment of statistical significance in this study.

Our findings suggest that virtual coaching may be a feasible and effective method for novice first‐year gastroenterology fellows to learn cognitive and technical EGD skills. Presently, there are limited resources available to comprehensively educate on endoscopy procedures.^18^ Standalone recorded procedure videos are available online for viewing, but this at best can only help with the cognitive portion of endoscopy training. The availability of SBML with VC would be able to bridge the gap for technical skills training.^19^

Virtual coaching can be applied broadly in endoscopy training. Even after the pandemic era, it can improve access to endoscopy training with experts and has the potential to provide high‐quality training to trainees from almost any location. In order for the SBML with VC training program to be more widespread and adaptable, industry partners and local institutions play crucial roles in ensuring the availability of simulation models and other infrastructures. Future randomized studies, with consistent clinical evaluations, to compare traditional apprentice‐based training with our SBML program with virtual coaching or direct coaching are needed to better understand the effectiveness of this method of training in endoscopy.

## CONFLICT OF INTEREST STATEMENT

Tonya R. Kaltenbach: Consultant for Verily Life Sciences and Research Support from Olympus. Roy M. Soetikno: Consultant for Olympus and Fujifilm. Amandeep Shergill: Consultant for Boston Scientific. The remaining authors declare no conflict of interest.
